# Interventional solutions for post-surgical problems: a lymphatic leaks review

**DOI:** 10.1186/s42155-024-00473-3

**Published:** 2024-08-10

**Authors:** Fernando M. Gómez, Tarik R. Baetens, Ernestos Santos, Boris León Rocha, Benjamín Horwitz, Sara Lojo-Lendoiro, Patricio Vargas, Premal Patel, Regina Beets-Tan, José J. Martínez-Rodrigo, Luis Martí Bonmatí

**Affiliations:** 1 Biomedical Imaging Research Group (GIBI2^30), La Fe Health Research Institute (IIS La Fe), Avenida Fernando Abril Martorell, València, 46026 Spain; 2 Radiology Department, La Fe University and Polytechnic Hospital, Avenida Fernando Abril Martorell, València, 46026 Spain; 3 Department of Radiology, The Netherlands Cancer Institute, Plesmanlaan 121, Amsterdam, 1066 CX The Netherlands; 4 Radiology, Division of Interventional Radiology, Memorial Sloan Kettering Cancer Center, New York, USA; 5 Department of Interventional Radiology, Hospital Clínico de la Universidad de Chile, Santos Dumont 999, Independencia, Región Metropolitana Chile; 6 Radiology Department, Facultad de Medicina Clínica Alemana-Universidad del Desarrollo, Santiago, 7650568 Chile; 7 Department of Radiology, Hospital Álvaro Cunqueiro, Estrada de Clara Campoamor, 341, Vigo, Pontevedra 36312 Spain; 8 Great Ormond Street Hospital for Children NHS Foundation Trust, Renal Unit, Level 7, Southwood Building, Great Ormond Street, London, WC1N 3JH UK

**Keywords:** Lymhangiography, Surgical lymphatic leak, Embolization

## Abstract

The lymphatic circulation plays a crucial role in maintaining fluid balance and supporting immune responses by returning serum proteins and lipids to the systemic circulation. Lymphatic leaks, though rare, pose significant challenges post-radical neck surgery, oesophagectomy, and thoracic or retroperitoneal oncological resections, leading to heightened morbidity and mortality. Managing lymphatic leaks necessitates consideration of aetiology, severity, and volume of leakage.

Traditionally, treatment involved conservative measures such as dietary restrictions, drainage, and medical management, with surgical intervention reserved for severe cases, albeit with variable outcomes and extended recovery periods. Lymphography, introduced in the 1950s, initially served as a diagnostic tool for lymphoedema, lymphoma, tumour staging, and monitoring chemotherapy response. However, its widespread adoption was impeded by alternative techniques like Computed Tomography, learning curves, and its associated complications. Contemporary lymphatic interventions have evolved, favouring nodal lymphangiography over pedal lymphangiography for its technical simplicity and reduced complexity.

Effective management of chylous leaks mandates a multimodal approach encompassing clinical evaluation and imaging techniques. In cases where conservative management proves ineffective, embolization through conventional lymphangiography by bipedal dissection or intranodal injection emerges as a viable option. This review underscores the importance of a comprehensive approach to diagnosing and treating lymphatic leaks, highlighting advancements in imaging and therapeutic interventions that enhance patient outcomes.

## Introduction

The lymphatic system (LS) is susceptible to be affected by various diseases, stemming from both malignant and benign origins. Among the primary causes of lymphatic leakages, we can find congenital anomalies and traumatic injuries affecting the LS [1]. Non-traumatic chyle leaks are frequently secondary to processes causing lymphatic obstruction, such as malignant occlusion, lymphatic vessel abnormality, systemic or congenital diseases, or idiopathic obstruction [2].

The most frequent causes of chyle leakage are iatrogenic injuries, mostly secondary to thoracic or abdominal surgery, with chylothorax rates as high as 3.9% after oesophagectomy [2] Typically, lymphatic leaks manifest in two scenarios: during surgical resections, particularly in oncological cases necessitating extensive lymphadenectomy, or in surgeries where the operative field intersects with major lymphatic vessels.

Traditionally, managing such complications leaned towards conservative measures [3]. However, persistent lymphatic leaks often prompted recourse to aggressive open or laparoscopic surgical interventions, albeit with associated morbidity, mortality, prolonged hospital stays and patient recovery periods [1, 3].

Initially, diagnostic procedures involved injecting blue dye between the toes, exposing a lymphatic vessel on the foot dorsum for cannulation after dissection, followed by ethiodized oil injection and serial radiography from the foot to the thorax to track contrast progression [3, 4].

Conventional lymphangiography (LA) emerged as a diagnostic tool in the 1950s, but with the advent of advanced imaging modalities such as ultrasound (US), computed tomography (CT), and magnetic resonance (MR), it was relegated to the background transitioning to a therapeutic modality with the introduction of thoracic duct (TD) embolization for chylothorax by Dr. Constantine Cope in the 90’s [3, 4]. During 2011 and 2012 intranodal lymphangiography surfaced as a simpler alternative [5–7]. Over recent decades, LA has ascended as a minimally invasive procedure, yielding favourable outcomes and witnessing technique refinements from pedal LA to encompass the treatment of diverse conditions like chylous ascites, paediatric chylothorax, plastic bronchitis, protein-losing enteropathy, lymphoceles, and thoracic duct stenting [8].

## Anatomy

**Fig. 1:**
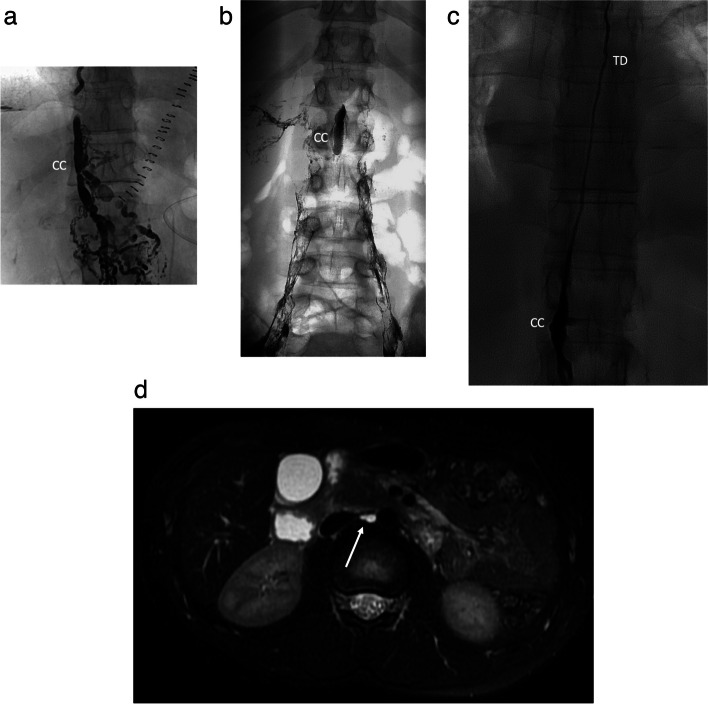
**a**-**d** The chyle cistern (CC) (**a**-**c**) and thoracic duct (TD) (**b**, **c**). The CC is usually located at the level of L1-L2, and it has a linear (**a**) or oval (**b**) shape. Sometimes the TD can be seen in T2-weighted sequences on MRI, arrow (**d**)

Besides the immune system’s transport network, the function of the LS is to return fluid and nutrients from interstitial tissues to the venous system. It is divided into three sections: soft tissue and extremities, hepatic and enteral lymphatics [9]. The hepatic and enteral lymphatics produce 80% of the lymphatic fluid, while the upper and lower extremities produce the remaining 20%. Lymph flows from the lower limbs, pelvis and abdomen to the Pecquet’s cistern or cisterna chyli (CC) and its continuation in the chest, the thoracic duct (TD) (Fig. 1a-d) [9, 10]. A smooth muscle layer composes the lymphatic duct walls and is susceptible to vagal, serotonin, norepinephrine, histamine and dopamine stimulation, increasing lymph flow, while opiates and octreotide decrease it [9].

**Fig. 2:**
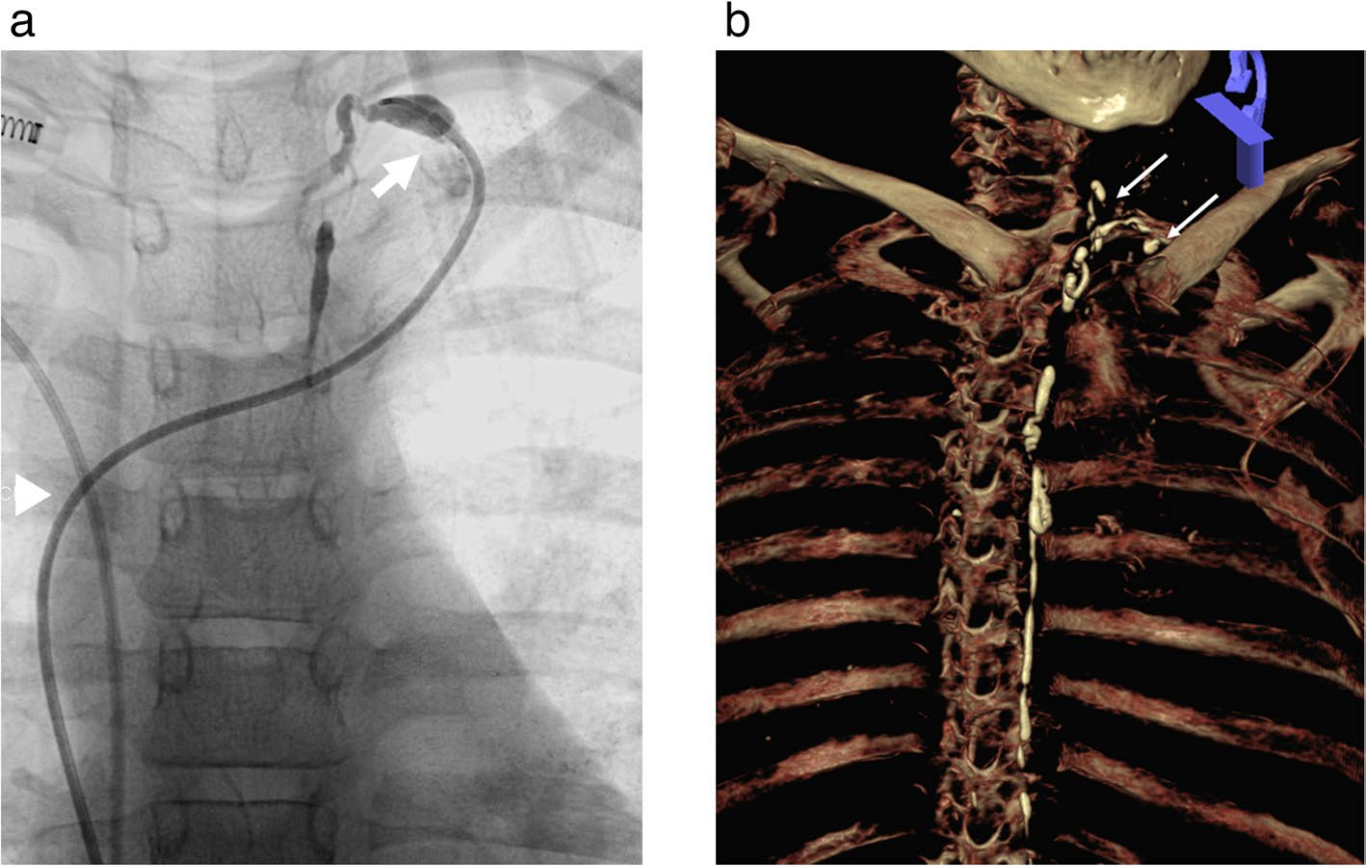
**a**, **b** Venolymphatic Anastomosis. **a** Retrograde catheterization with an angiographic catheter (arrow head) of the TD showing a single anastomosis (single arrow). **b** 3D Computed Tomography reconstruction in a TD junction showing multiple anastomosis (multiple arrows)

The CC is commonly located between L1 and L2, parallel to and behind the inferior cava vein, joining the TD in the upper third of the abdomen, deep to the arcuate ligament. It is present in 65% to 85% of patients and its shape is variable: oval (50%), linear (40%) or round (10%) [7] (Fig 1a, b). It can measure up to 5 cm in length and average diameter of 6-7 mm [9]. The intestinal, hepatic and lumbar trunks drain the lymph/chyle into the CC. Cephalad and in the chest, the TD is located parallel and to the right of the oesophagus, crossing to the left at the approximate level of the middle third of the thoracic vertebral bodies, where it lies anterior to the intercostal branches of the thoracic aorta. Then, it rises to the dome pleura and drains into the left subclavian vein, next to the jugular subclavian confluence (Fig. 1d). TD mesures approximately 45 cm in length and 2-5 mm in diameter. The anatomy of the TD and CC is variable (normal anatomy in 40-60% of the patients) with a variable crossing level, number of thoracic collaterals (which increase surgical complexity) and the anatomy of venous-lymphatic anastomosis with the left subclavian vein (single trunk with a big caliber or several small vessels) (Fig. 2a, b). These anatomic variants should be carefully considered when planning a retrograde embolization [11].

## Technical approach

LA is a minimally invasive radiological technique in which the contrast is injected into the LS after puncturing a duct or a lymph node with a thin needle (usually thinner than 24G). The preferred contrast medium is lipiodol, but other agents such as water-soluble iodinated contrast or gadolinium can also be applied mainly for diagnostic purposes on CT or MR scans [12]. Ethiodized oil (lipiodol®, Gerbet…) is a lipid based iodinated contrast, consisting of iodine molecules combined with fatty acids from poppy seed oil, which generates a continuous column of contrast due to its chemical properties. Interestingly, it can cause in some cases sclerosis and occlusion of the leak achieving a therapeutic effect, this happens between 50% and 75% of the cases being more frequent when the volume of the leak is below 500cc/24h [13–15].

The recommended maximum total dose should not exceed 20mL. An appraisal of a suitable volume has been proposed at a maximum dose of 0.25 ml/kg [16]. However, according to the authors experience, when a leak is clearly present, use of higher doses can be reasonable as most of the lipiodol will not be delivered to the venous system. Once injected into the LS, contrast follows the lymph course from extremities towards pelvis, abdomen and finally thorax.

**Fig. 3:**
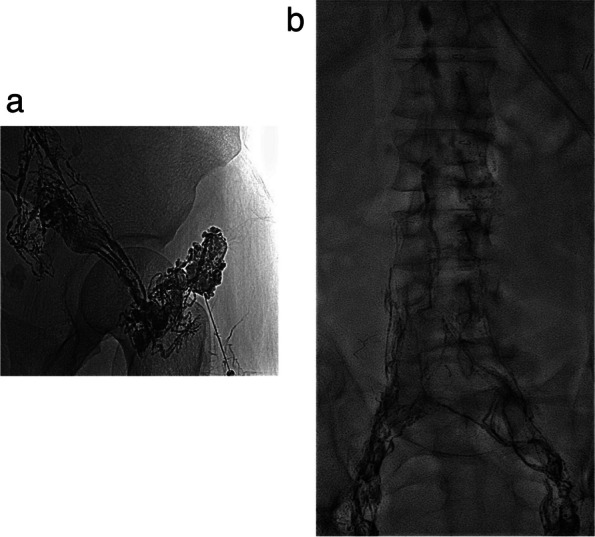
**a**, **b** Transnodal lymphangiography showing filling of inguinal lymphatic vessels and lymphatic nodes (**a**). Approximately 30 minutes after the beginning of the injection iliac lymphatic vessels and nodes are depicted (**b**)

Conventional bipedal LA is regarded as a complex procedure due to the intricate steps involved. The interventionist is required to identify and isolate a lymphatic vessel on the foot after administering a dye (0.5 cc of methylene blue or blue Isosulfan 1%) into the web spaces of the toes. Once the operator identifies the staining of the lymphatic vessels, a dissection is made on the dorsum of the foot to find the access into the vessel with a fine needle (27 gauge - 30 gauge) [4, 17]. X-ray images of the extremity are planned every 15 min, while for the abdomen it may require up to 2-5 hrs. Recently, intranodal LA has emerged as a simpler and less invasive alternative to traditional bipedal LA. It consists of a lymph node ultrasound-guided percutaneous puncture, usually in the inguinal region (potentially can be performed on any lymph node), with then a direct injection of the contrast medium (Fig. 3). This technique is easier than the classic approach, makes diagnostic LA compatible with the basic skills of any interventional radiologist and decreases the time duration of the study [5]. With this method, it may take than 1h for the contrast to reach the abdomen [4]. It has been reported a technical success rate of up to 87% for IL [16–19]. Although the evidence supporting this access is recent, its first description was made in 1930 by Funaoka [20]. The technical objective is to precisely position the tip of a fine needle (ranging from 23G to 26G), ideally at the corticomedullary junction. Then the needle is connected to a variable volume syringe or a pump and the contrast is injected at a rate of 5-10 mL/h under intermittent fluoroscopic vision after confirming the correct progression of the contrast column. If the location of the needle is correct, the operator could see opacification of the lymph node and its efferent lymphatic ducts. If contrast flows out of the lymph node, the needle must be repositioned unless minimal extravasation happens and clear progression of lipiodol is confirmed. When performing this technique on the groin, the same process can be repeated in the contralateral side to shorten the duration of the procedure [4–6].

The calculated total dose of contrast must be divided into the number of lymph nodes to be punctured. Lipiodol injection can be continued until the maximum dose to get a greater embolic effect. If the total dose is injected before reaching the third lumbar body level or the desired level (i.e., the thoracic duct), then the operator may facilitate the spread of the contrast administering 1 mL/5 min saline [9]. Warming the lipiodol can help to achieve higher speed of contrast injection since old lymphangiography pumps heated lipiodol while injecting. To evaluate lipiodol circulation, the operator can observe the lymphatic distribution with fluoroscopic vision, immediately after its injection and at 1, 3, 5, 10, 15, 30, and 45 min (Fig. 3b). According to the speed of distribution of contrast and in order to find the lymphatic leak, in cases when it is not visualised during the procedure, the operator can complete the study with abdominal and chest radiographs in 12-24hrs, or CT or MRI. Both of these methods have also been employed to assess a patient's response to therapy [9, 17]. Once the leak has been demonstrated, physicians should decide its management. The first to describe a successful percutaneous treatment for chylothorax was Cope in 1998 [3]; later, lipiodol lymphangiography and transnodal glue embolization were used to treat pelvic leaks [21, 22]. Once the TD and/or CC are depicted, then it is possible to perform selective catheterization and embolization, as described in the following methods:

### Antegrade percutaneous transabdominal access

**Fig. 4:**
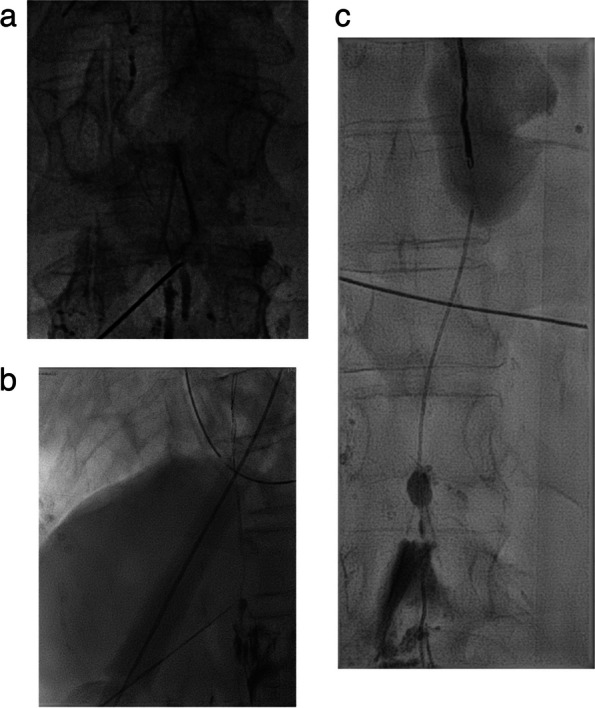
**a**-**c** CC percutaneous transabdominal anterograde fluoroscopy guided access using a 22 G needle (**a**). Insertion of a 0,018 ´ ´ guidewire in the TD through the CC (**b**). Progression of a 2.4 microcatheter and subsequent coiling as scaffold for glue embolization (**c**)

A fine needle (21G-22G, measuring 15cm-20cm in length) guided by fluoroscopy is utilised for the procedure. The objective is to puncture the CC. If the CC is not identified, an alternative is to puncture the abdominal portion of the TD. Subsequently, a microwire (0.014" or 0.018") is inserted into the CC or TD, followed by the advancement of a microcatheter. If the leak can be identified at this stage, the operator should aim for maximal selectivity before proceeding to inject the embolic agent (Fig. 4a). In certain scenarios, a through-through technique may be employed to enhance stability and effectively embolize leaks, particularly those located at lower levels, such as abdominal leaks.

### Retrograde catheterization from the subclavian vein

TD can be accessed at the level of its anastomosis with the subclavian vein, either after intravenous access or by direct puncture guided by ultrasound or fluoroscopy when it is opacified [23]. This method may be difficult if the size of the lymphatic vessel or vessels forming the anastomosis are small or in cases where a complete postoperative section of the TD occurred [8] (Fig. 2). Balloon-occluded endolymphatic retrograde abdominal lymphangiography (BORAL) and embolisation (BORALE) are novel techniques used for the diagnosis and treatment of chylous ascites. These techniques use an occlusion balloon placed into the TD to prevent reflux of sclerosing material when diagnosing and treating abdominal lymphatic leaks [24]. Another option is to perform US guided direct puncture of the thoracic duct close to the subclavian level**.** The thoracic duct can be accessed after lipiodol lymphangiography or by means of ultrasound or fluoroscopic guidance [25–27]. Once access is established, the embolization begins.

## Indications

LA, either alone or in combination with embolization, serves as a viable diagnostic and/or treatment option for conditions such as chylothorax, chylous ascites, lymphoceles, and chylopericardium.

## Chylothorax

Chylothorax is the presence of lymphatic or chylous fluid in pleural space. Milky pleural fluid with a triglyceride concentration greater than 110 mg/dl or the presence of chylomicrons on lipoprotein electrophoresis confirms the diagnosis of chylothorax [28]. The most common cause is due to iatrogenic trauma during surgery, chylothorax has been reported in 0.42% of cases of thoracic surgery [29]. Non-traumatic causes have also been described, with malignant disorders being the most frequent, especially in patients affected by lymphoma or pulmonary cancer. As a consequence of invasion or compression by the tumour, the pressure in the lymph vessels increases, making them more susceptible to lymphatic extravasation. Other causes are idiopathic, congenital, autoimmune or infectious (mainly tuberculosis) [11, 14, 15].

Management of chylothorax depends on its severity. Chylothorax is considered to be low output when there is a loss of less than 500 cc per day, and it can be treated conservatively, with a low-fat diet, parenteral nutrition, octreotide administration and pleural drainage tubes. These conservative measures are successful in up to 50-60% of the cases [30].

High output chylothorax is defined when there is loss of more than 1000 cc of lymph in 24h; and often is not controlled with conservative measures. In these cases, patients suffer loss of nutrients, immune cells and proteins, resulting in a high morbidity and mortality [15]. Traditionally it has been approached surgically by attempting TD ligation by means of video assisted thoracoscopic surgery (VATS). Conversion to open thoracotomy is performed when it is not possible to locate the TD thoracoscopically but it is associated with higher morbidity and mortality rates (2.1% vs. 38.8%respectively) [31]. Reported surgical success rates are 70% in children [7] and range between 70% and 100% in adults [32]. Other surgical alternatives are the creation of a pleuroperitoneal shunt and pleurodesis.

LA, with or without TD embolization, offers a less invasive approach supported by growing evidence demonstrating favourable outcomes in both adult and paediatric populations, including patients that recurred post-surgery [11, 15]. Decision-making typically hinges on individual patient evolution following conservative interventions when chyle output falls within the range between two breakpoints (> 500cc and <1000cc/day) [2] (Fig. 4b, c).

## Chylous ascites

Similar to chylothorax, the aetiology of chylous ascites can be traumatic or non-traumatic. Non-traumatic causes include inflammatory and infectious diseases like tuberculosis, filariasis, radiation, pancreatitis neoplasms as lymphoma traumatic or postoperative causes encompass blunt abdominal trauma, aortic surgery and retroperitoneal lymphadenectomy, among others [33, 34]. Typically, management commences with a conservative approach. In recurrent or severe cases, surgical alternatives may be considered.

**Fig. 5:**
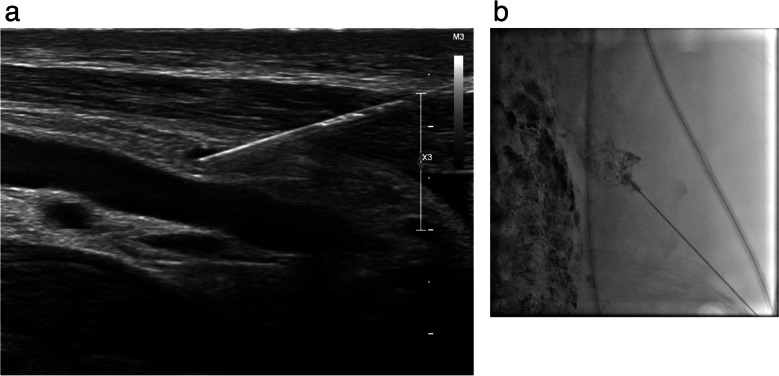
**a**, **b** Ultrasound guided puncture of a mesenteric lymph node (**a**) and mesenteric lymphangiography (**b**)

**Fig. 6:**
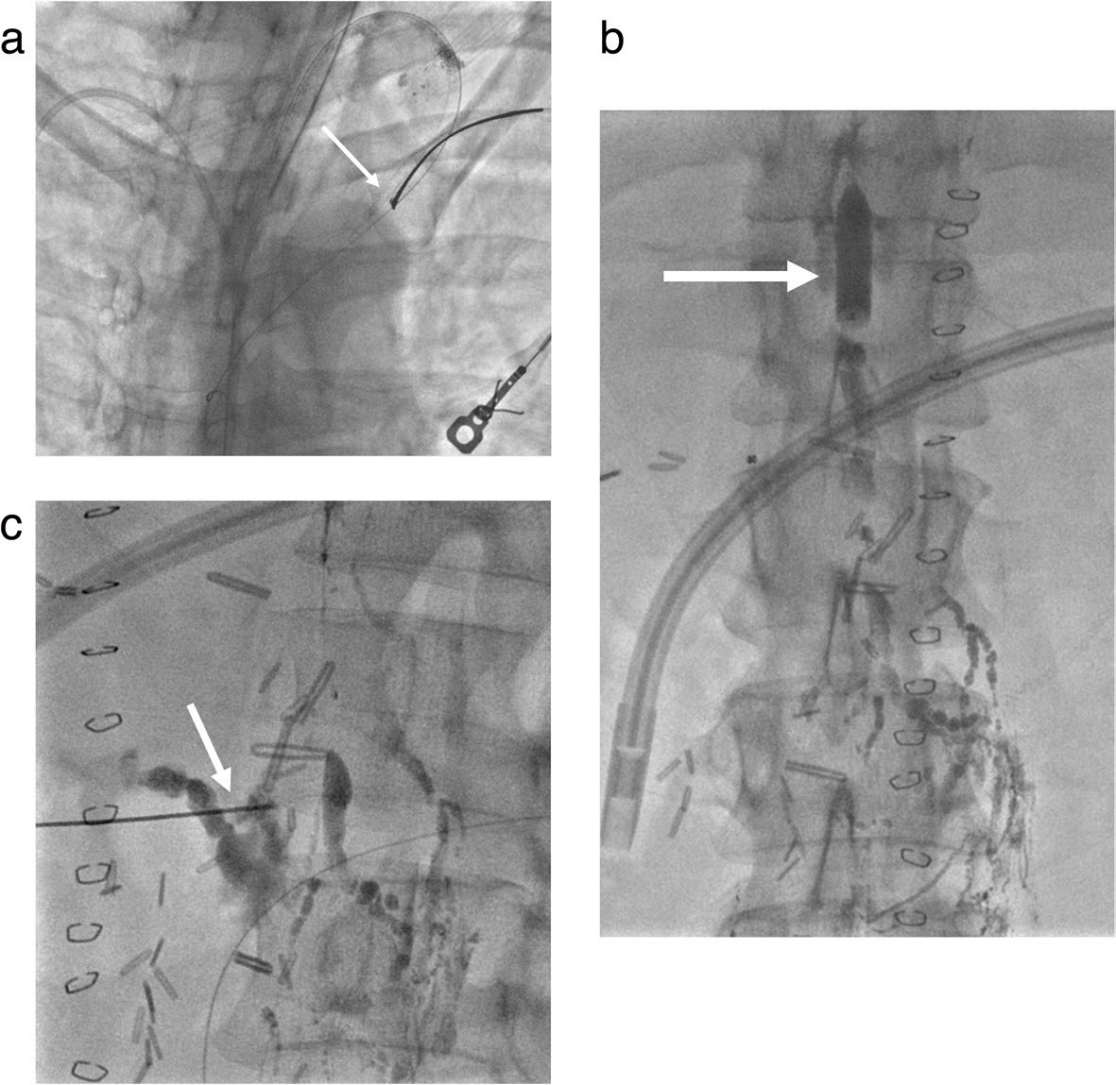
**a**-**c** Tansabdominal access through the CC and wire snearing (arrow) via subclavian vein approach (**a**). Balloon inflated at T12 L1 level (arrow) Retrograde injection of contrast with demonstration of the leak at the pancreatic bed (**b**). Percutaneous embolization (arrow) with glue (**c**)

**Fig. 7:**
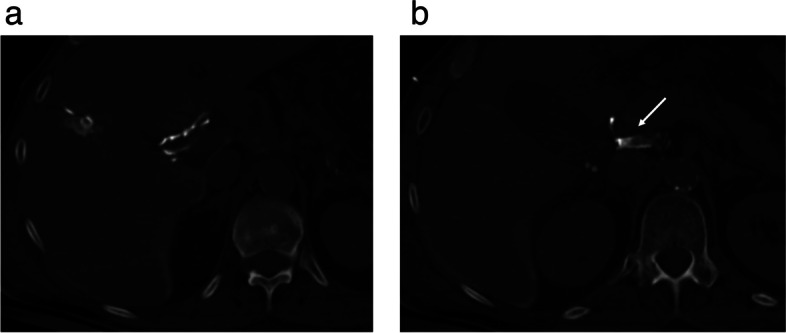
**a**-**c** Transhepatic Computed Tomography lymphangiography (**a**) showing a lymphatic leak (arrow) (**b**) and subsequent embolization using Glue (1:3) (**c**)

Despite interventional procedures have been documented in the management of chylous ascites, the effectiveness is lower than thoracic duct embolization for chylothorax. Approximately 55% of patients with chylous ascites have lipiodol extravasation detected with LA which makes more difficult to define the best treatment strategy. The success of LA and embolization is closely tied to the accurate representation of the leak [34]. Several percutaneous procedures have been described in this clinical setting including TD embolization, BORALE technique, mesenteric lymphangiography and interstitial embolization [10, 24, 34]. When lymphatic leak is not demonstrated following groin lymph node injection direct puncture of a mesenteric lymph node can be beneficial (Fig 5a, b). Direct access to the leak, along with catheterization and embolization, or retrograde access with balloon occlusion and embolization, may also yield effective occlusion of the leak [24, 35] (Fig. 6a-c). Furthermore, in certain clinical scenarios, such as chylous ascites post-pancreatic or liver surgery, transhepatic lymphatic embolization has demonstrated efficacy in treating the leak [36–38] (Fig 7a-c).

## Pelvic leaks

Lymphocele is defined as the presence of a collection of lymph surrounded by a capsule without epithelium. In the pelvis and groin, lymphatic leakage occurs most frequently after renal transplantation, surgeries affecting the vascular bundle and lymphadenectomy performed after prostatectomy or other surgeries in the pelvis [21].

The main surgical technique described is an open drainage and marsupialisation. On the other hand, interventional techniques include percutaneous drainage and sclerotherapy. This involves drainage catheter insertion into the collection under image guidance (US, CT or fluoroscopy) and injection of a sclerosant agent for a variable period of time during collection, which is then removed [39]. There are different agents described in literature: among others are doxycycline, bleomycin, sodium tetradecyl sulphate, ethanol or absolute alcohol, povidone iodine and tetracycline [39–41]. Although this treatment is sometimes successful after one session, based on the current evidence, the average number of sclerotherapy procedures to remove the drain ranges from 1 to 4.

**Fig. 8:**
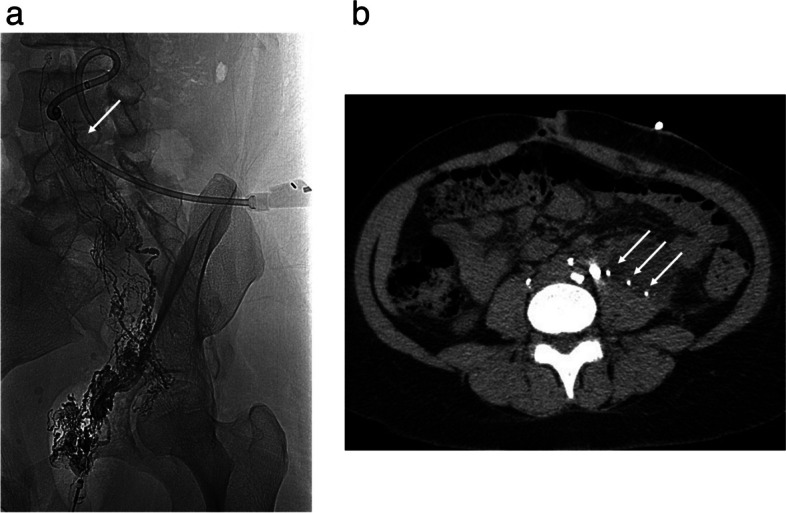
**a**, **b** Transnodal lymphangiography in a retroperitoneal lymphocele showing Lipiodol leak (arrow) (**a**). Computed tomography depicting Lipiodol droplets inside the lymphocele (multiple arrows) (**b**)

**Fig. 9:**
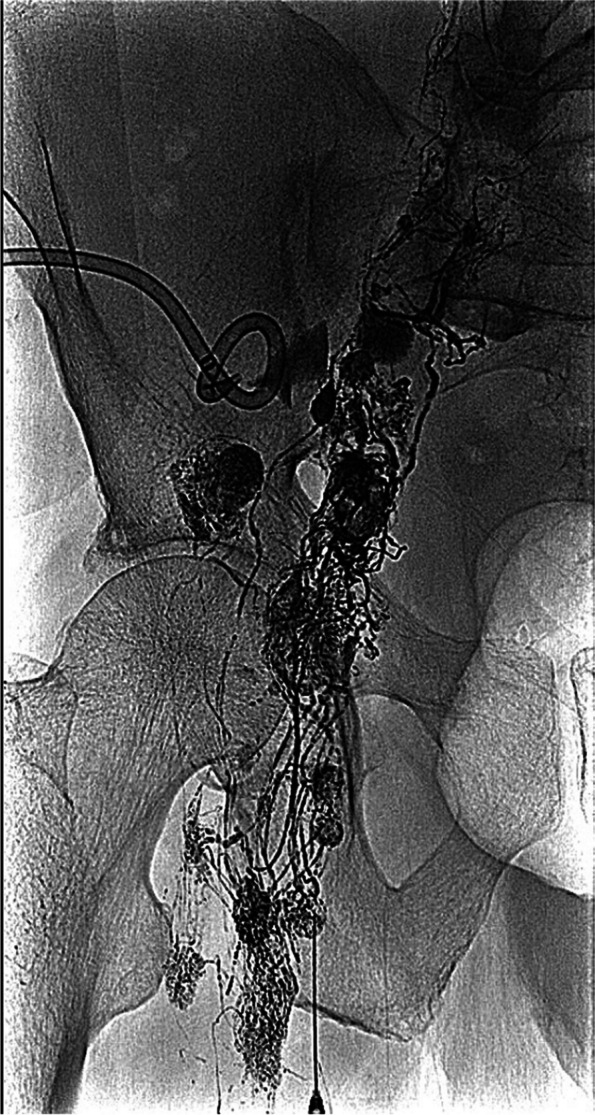
Lymphangiography showing a lymphatic leak in a post-trasplant lymphocele (arrow)

**Fig. 10:**
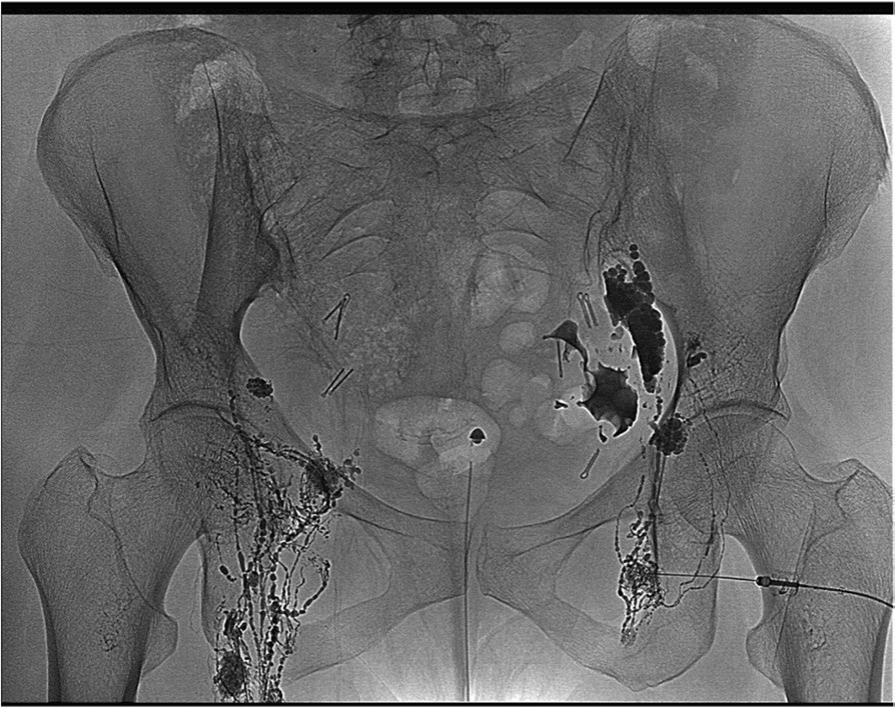
Massive left lymphatic leak after bilateral retroperitoneal iliac lymphadenectomy embolized by beans of glue 1:3

In cases of severe leakage or recurrence, it is possible to identify the direct leak from a lymphatic duct. In this context, LA has been recommended for diagnosis of possible leaks and their treatment [21, 22] (Fig. 8a, b). Several authors have proposed a mixed treatment, prioritising a minimal invasive approach as the first measure. Hamza et al [42] described a treatment algorithm for post-renal transplant lymphocele, with percutaneous treatment performed as the first option and, in cases of recurrence or difficult access, surgical treatment (Fig 9). For these patients, or those with lymphatic leakage after surgery, interstitial lymphatic embolisation by direct nodal puncture and glue injection (N-CBA mixed with lipiodol 1:3 or 1:4) has also been described as an effective treatment and it may offer a shorter time to drain removal compared to sclerotherapy [43] (Fig 10).

## Complications

The complication rate following LA and lymphatic embolization ranges from 0% to 3% [8, 14, 16]. The main risk of using lipiodol in LA is non-target embolization to lungs or brain. The underlying mechanism for how lipiodol reaches the cerebral circulation may involve right-to-left intracardiac shunt so previously performing an echocardiogram is recommended [35]. Other reported complications include the alteration of thyroid function in patients with hyper or hypothyroidism and impaired renal function [16].

Embolization of non-target territories, particularly the pulmonary arteries, stands out as one of the most frequently reported complications [14]. Lipiodol embolization in the cerebral arterial territory leading to secondary cerebral stroke has also been described [15, 39]. Other potential complications may include bleeding or infection associated with percutaneous access. Embolization to the portal territory has also been documented following transhepatic approach. Furthermore, Lipiodol use has been associated with alterations in thyroid function in patients with hyper or hypothyroidism and impaired renal function [16]. Delayed complications following thoracic duct embolization can have an overall incidence rate as high as 14.3% and may include chronic diarrhoea, chronic leg swelling, and abdominal swelling [34].

## Discussion

Lymphatic leaks and effusions can arise from congenital conditions or traumatic injuries to the LS. Typically, surgical management of these leaks entails complex and aggressive interventions, often struggling to pinpoint the source of the problem. Traditional bipedal LA presents itself as a cumbersome technique widely utilised for lymphatic system evaluation and tumour staging prior to the emergence of cross-sectional imaging. However, the advent of CT and MRI led to a decline in the use of LA and a gradual disappearance of pedal dissection from the repertoire of radiologists, contributing to its diminished utilisation.

The development of intranodal LA and various percutaneous approaches has streamlined the technique, rendering it more accessible to interventional radiologists of varying skill levels. Nevertheless, with the availability of less invasive diagnostic imaging techniques like dynamic MRI lymphangiography, which not only evaluates the origin of a lymphatic leak but also facilitates therapeutic planning [44], lipiodol LA should be reserved for cases necessitating immediate treatment within the same procedure.

However, published evidence is primarily derived from retrospective studies, each employing different treatment protocols and a variety of embolic agents. Consequently, comparing the efficacy of these agents proves challenging. Reported success rates vary widely, ranging from 74% to 100% [24–27]. The assortment of embolic agents utilised includes coils, ethylene vinyl alcohol (EVOH), cyanoacrylates, alcohol, either alone or in combination [8, 17, 20]. For glue embolization, described dilutions typically range between 1:2 or 1:2.5 [13, 15]. Notably, Itkin et al. advocate for using coils as a scaffold to support subsequently injected glue, thus minimising the risk of unintended embolization [17]. They also recommend using glue alone in cases where coil embolization is hindered by limited space. In situations where cannulation of the TD or CC is not feasible, CC disruption can be performed under fluoroscopic guidance or computed tomography vision [8, 15, 27].

Management should adopt a multidisciplinary approach, commencing with conservative treatment and escalating to more invasive alternatives only in cases of treatment failure. Given the relatively low number of patients with lymphatic complications and the technical complexity of cases requiring embolization, centres with ample experience should undertake LA and percutaneous embolization for lymphatic leaks in the pelvis, abdomen, or thorax.

Although there are no trials comparing the effectiveness of surgical repair versus lymphatic embolization, the efficacy of LA and embolization, coupled with their low rate of major complications, makes it ethically questionable to conduct such trials. As a result, surgery should be reserved solely for cases where percutaneous embolization proves unsuccessful.

## Conclusions

LA and embolization should be considered the treatment of choice for complex postsurgical lymphatic leaks after failure of conservative treatment. It is important that these treatments are managed multidisciplinarity and performed in experienced centres, evaluating minimally invasive treatment options to accelerate the patient's recovery and potentially improve results.

## Data Availability

NA.

## Abbreviations

LS: Lymphatic system
LA: Lymphangiography
CT: Computed tomography
MR: Magnetic Resonance Imaging
CC: Chili cistern
TD: Thoracic duct
EVOH: Ethylene vinyl alcohol
