# Correction: Interventional solutions for post‑surgical problems: a lymphatic leaks review

**DOI:** 10.1186/s42155-024-00483-1

**Published:** 2024-09-17

**Authors:** Fernando M. Gómez, Tarik R. Baetens, Ernestos Santos, Boris Leon Rocha, Benjamin Horwitz, Sara Lojo‑Lendoiro, Patricio Vargas, Premal Patel, Regina Beets‑Tan, Jose J. Martinez‑Rodrigo, Luis Marti Bonmati

**Affiliations:** 1 Biomedical Imaging Research Group (GIBI2^30), La Fe Health Research Institute (IIS La Fe), Avenida Fernando Abril Martorell, València, 46026 Spain; 2 Radiology Department, La Fe University and Polytechnic Hospital, Avenida Fernando Abril Martorell, València, 46026 Spain; 3 Department of Radiology, The Netherlands Cancer Institute, Plesmanlaan 121, Amsterdam, 1066 CX The Netherlands; 4 Radiology, Division of Interventional Radiology, Memorial Sloan Kettering Cancer Center, New York, USA; 5 Department of Interventional Radiology, Hospital Clinico de la Universidad de Chile, Santos Dumont 999, Independencia, Región Metropolitana, Chile; 6 Radiology Department, Facultad de Medicina Clinica Alemana- Universidad del Desarrollo, Santiago, 7650568 Chile; 7 Department of Radiology, Hospital Álvaro Cunqueiro, Estrada de Clara Campoamor, 341, Vigo, 36312 Pontevedra Spain; 8 Renal Unit, Great Ormond Street Hospital for Children NHS Foundation Trust, Level 7, Southwood Building, Great Ormond Street, London, WC1N 3JH UK

**Correction: CVIR Endovasc 7**,** 61 (2024)**

10.1186/s42155-024-00473-3

Following publication of the original article [1], the author reported that the affiliations 3 and 4 have been interchanged. The original article has been corrected.

The affiliations 3 and 4 currently read:

3 Radiology, Division of Interventional Radiology, Memorial Sloan Kettering Cancer Center, New York, USA.

4 Department of Radiology, The Netherlands Cancer Institute, Plesmanlaan 121, Amsterdam 1066 CX, The Netherlands.

The affiliations 3 and 4 should read:

3 Department of Radiology, The Netherlands Cancer Institute, Plesmanlaan 121, Amsterdam 1066 CX, The Netherlands.

4 Radiology, Division of Interventional Radiology, Memorial Sloan Kettering Cancer Center, New York, USA.
